# Acute effects of a single functional neurology session on autonomic modulation in a patient with mild depression: a case report

**DOI:** 10.3389/fnins.2025.1567062

**Published:** 2025-05-23

**Authors:** Vicente Javier Clemente-Suárez, Guillermo Escribano-Colmena, Eduardo Navarro-Jiménez, Jorge Rey-Mota

**Affiliations:** ^1^Faculty of Sports Sciences, Universidad Europea de Madrid, Madrid, Spain; ^2^Independent Researcher, Boadilla del Monte, Spain; ^3^Facultad de Ciencias de la Salud, Universidad Simón Bolívar, Barranquilla, Colombia

**Keywords:** functional neurology, heart rate variability, autonomic regulation, depression, stress

## Abstract

Autonomic dysregulation is frequently observed in individuals with psychological distress, including mild depression, anxiety, loneliness, and chronic pain. Functional neurology offers a non-invasive, sensorimotor-based approach aimed at restoring autonomic balance through targeted neural stimulation. This case report explores the acute effects of a single-session multimodal functional neurology intervention on heart rate variability (HRV) in a 38-year-old female patient presenting with mild depression, moderate anxiety, high loneliness, psychological inflexibility, and chronic musculoskeletal pain. Baseline psychological assessments were conducted to characterize the patient’s emotional and cognitive profile, but no post-intervention psychological data were collected. HRV was measured before and after the intervention using photoplethysmography, with time and frequency domain analyses performed. Post-intervention results showed an increase in high-frequency (HF) power and a decrease in low-frequency (LF) power, suggesting enhanced parasympathetic modulation. These findings provide preliminary physiological evidence that a brief neuromodulatory intervention may positively influence autonomic function. Further controlled research is warranted to assess the consistency and clinical significance of these effects.

## 1 Introduction

In recent years, the prevalence of psychological disorders has increased significantly, with anxiety, depression, and stress-related conditions becoming more widespread (Carreira-Míguez et al., 2022; Merino del Portillo et al., 2024). This rise can be attributed to multiple factors, including social and economic pressures, urbanization, and lifestyle changes that contribute to the mental health burden (Patel et al., 2018; Clemente-Suárez et al., 2021a). Global data indicate a sharp uptick in the diagnosis and treatment of mental health disorders, highlighting an urgent need for effective interventions. The growing awareness and reduction of stigma surrounding mental health have also played a role in this increased reporting, allowing for more accurate estimations of psychological distress across diverse populations (Baxter et al., 2013; Clemente-Suárez et al., 2021b).

Autonomic dysregulation has garnered increasing attention in recent research due to its role in both psychological and physiological disturbances. While this relationship is well-documented, the primary focus of this study is to evaluate the immediate effects of a single functional neurology intervention on heart rate variability (HRV), a marker of autonomic modulation (Thayer and Lane, 2009). Psychological assessments were included to provide context on the participant’s distress levels but were not intended to establish causal relationships between autonomic and psychological parameters (McEwen, 2017). Heart rate variability (HRV), a key marker of autonomic balance, has emerged as a useful tool for assessing ANS function in patients with psychological disorders. Studies consistently show that individuals with anxiety and depression tend to exhibit lower HRV, reflecting reduced parasympathetic activity and heightened sympathetic dominance (Chalmers et al., 2016). These findings highlight the importance of targeting autonomic regulation in therapeutic interventions for psychological conditions. Although this study explores potential links between functional neurology interventions and autonomic modulation, the findings are heuristic in nature and aim to provide preliminary insights into these relationships.

Recent research into non-invasive neuromodulation have provided preliminary support for its role in autonomic and emotional regulation. Case reports and small-scale studies have suggested that vestibular stimulation may modulate brainstem-autonomic circuits, influencing heart rate variability and stress-related outcomes (Hammam and Macefield, 2017). Likewise, transcutaneous vagus nerve stimulation has shown potential in enhancing parasympathetic activity and alleviating symptoms of depression and anxiety (Fang et al., 2016). These findings offer a neurophysiological basis for combining such approaches within functional neurology protocols to address both emotional dysregulation and autonomic dysfunction. Building upon this framework, the present case study investigates the immediate impact of a multimodal neuromodulation session grounded in functional neurology on heart rate variability in a patient with mild depression.

In this line, lasts advancements in functional neurology have introduced innovative approaches to treating organic and neurological dysfunctions through non-invasive sensory stimulation protocols. Functional neurology techniques, particularly those targeting neurological imbalances, have shown promise in modulating autonomic function and improving well-being. For example, a single-session functional neurology intervention applying stimulation to the semicircular canals led to immediate improvements in autonomic modulation and neuromuscular control in individuals with vestibular dysfunctions, as evidenced by changes in heart rate variability and symmetry in saccadic responses (Escribano-Colmena et al., 2025a,b). Similarly, interventions directed at vestibulo-ocular reflex dysfunction demonstrated acute psychophysiological effects, including improved performance in postural and visual tasks, highlighting the role of brainstem-level neuromodulation (Escribano-Colmena et al., 2025a,b). Moreover, functional neurology has shown effectiveness at the muscular level; for instance, a recent study observed that a single intervention on neuromuscular trigger points induced significant increases in pressure pain thresholds and thermal responses, suggesting modulation of sensorimotor integration and peripheral autonomic activity (Rey-Mota et al., 2025a,b). These findings reinforce the hypothesis that brief, targeted neuromodulatory interventions may acutely enhance communication between the brain and autonomic nervous system.

Therefore, the main objective of this case study was to explore the acute effects of a single-session multimodal functional neurology intervention on autonomic modulation, as measured by heart rate variability (HRV), in a female patient with mild depression and associated psychological symptoms. Although the participant’s emotional profile—including depression, anxiety, loneliness, and psychological inflexibility—was assessed at baseline to contextualize autonomic status, the intervention was designed to target physiological regulation exclusively. The initial hypothesis was that a single session of functional neurology would produce measurable changes in HRV, specifically an increase in high-frequency (HF) power, reflecting enhanced parasympathetic activity, and a decrease in low-frequency (LF) power, reflecting reduced sympathetic modulation. This case was approached as an exploratory, hypothesis-generating study aiming to provide preliminary physiological data on the potential autonomic effects of functional neurology techniques. The central hypothesis of this study was that a single-session multimodal functional neurology intervention would produce acute changes in autonomic regulation, as evidenced by HRV parameters. Specifically, it was expected that the intervention would lead to an increase in HF power—reflecting enhanced parasympathetic activity—and a decrease in LF power—associated with reduced sympathetic dominance. Other clinical characteristics such as depression, chronic pain, and emotional distress were documented as contextual background but were not considered outcome variables. This case study was designed as a hypothesis-generating exploratory analysis within a broader research line, recognizing the limitations inherent to single-subject experimental designs while aiming to provide preliminary evidence to support future controlled trials.

## 2 Methods

### 2.1 Participant

This single-case study involved a 36-year-old female with chronic pain (6 months, 8/10 intensity) and mild psychological distress, meeting inclusion criteria for chronic pain, autonomic dysregulation (reduced baseline HRV values compared to normative references), and mild to moderate psychological distress based on Zung Depression Scale, STAI, and PSS-4 scores. Exclusion criteria ruled out severe psychiatric disorders, medications affecting autonomic function, recent neuromodulatory therapies, or neurological/systemic illnesses. The participant, identified through clinical screening, represented a case of mild autonomic dysregulation and psychological distress, aligning with the study’s objectives. These characteristics were considered part of the patient’s baseline psychological and somatic profile, used to contextualize her autonomic state. However, no post-intervention measures of pain or emotional symptoms were collected, and they were not defined as outcome variables. The focus of the study was limited to the analysis of autonomic modulation through heart rate variability (HRV) before and after the intervention. The study adhered to the Helsinki Declaration, with ethical approval (code 2024–739) and informed consent obtained, ensuring the participant’s rights and awareness of study details.

### 2.2 Study design

This single-case study followed a pre-post observational design to evaluate the immediate autonomic response to a functional neurology intervention. All procedures were completed in one session under controlled conditions. The timeline included an initial resting HRV recording, the neuromodulation intervention, and a follow-up HRV recording immediately after treatment.

The protocol was conducted in a quiet clinical room, maintaining consistent lighting, temperature, and minimal auditory distractions to reduce external influence on autonomic activity. The participant remained seated and relaxed throughout the measurements and was instructed to breathe naturally without speaking or moving.

The intervention, lasting approximately 20 min, was individualized based on the patient’s functional neurological findings and followed established sensorimotor stimulation protocols. No other treatments or physical activity occurred between the baseline and post-intervention assessments. The aim was to isolate the acute physiological effects of the intervention and detect any immediate changes in HRV metrics under comparable pre-post conditions.

### 2.3 Psychological assessment

A comprehensive battery of validated psychological questionnaires was administered 1 day before the intervention to evaluate the participant’s baseline emotional and cognitive-affective status. All instruments were used in their Spanish-language validated versions to ensure cultural and linguistic appropriateness.

Personality traits were measured using a shortened Spanish version of the Big Five Inventory (BFI-10), which demonstrated adequate internal consistency (Cronbach’s α = 0.81) (Rammstedt and John, 2007; Rodriguez-Besteiro et al., 2021). This tool assessed the core dimensions of personality, including extraversion, agreeableness, conscientiousness, neuroticism, and openness to experience.

The Zung Self-Rating Depression Scale (SDS) was employed to measure depressive symptoms. This 20-item scale uses a 4-point Likert response format, with total scores ranging from 20 to 80. Scores of 50 or above are indicative of mild to severe depressive symptoms (Cronbach’s α = 0.84) (Zung, 1965).

Loneliness was assessed using the UCLA Loneliness Scale – Short Form, which demonstrated high internal consistency (Cronbach’s α = 0.87). Scores of 6–8 were interpreted as moderate loneliness (Russell, 1996; Rodriguez-Besteiro et al., 2023).

Anxiety was assessed with the State–Trait Anxiety Inventory – Short Form (STAI-SF), a 6-item scale evaluating state anxiety. Items are rated on a 4-point Likert scale, yielding a total score ranging from 6 to 24. This version has shown strong internal consistency in Spanish-speaking populations (Cronbach’s α = 0.86–0.95) (Van Knippenberg et al., 1990).

The Acceptance and Action Questionnaire II (AAQ-II) was used to evaluate psychological inflexibility, a transdiagnostic process related to experiential avoidance. The AAQ-II includes 7 items rated on a 7-point Likert scale and has demonstrated robust psychometric properties (Cronbach’s α = 0.84–0.94) (Bond et al., 2011).

Finally, perceived stress was assessed using the Perceived Stress Scale-Short Form (PSS-4). This 4-item scale assesses the degree to which situations in one’s life are perceived as stressful, with acceptable internal reliability (Cronbach’s α = 0.72) in Spanish samples (Vallejo et al., 2018).

Although a comprehensive psychological assessment was conducted prior to the intervention to characterize the participant’s baseline profile, no post-intervention psychological measures were collected. The outcome evaluation was limited to physiological changes in HRV, and no follow-up assessments of mood, pain, or cognitive-emotional variables were performed. This decision was made to isolate the immediate autonomic response to the neuromodulatory protocol.

### 2.4 Autonomic modulation assessment

HRV was measured using a validated photoplethysmographic sensor (emWave Pro Plus^®^, HeartMath Inc., Boulder Creek, CA, USA), which has demonstrated high correlation with electrocardiographic methods for time and frequency domain parameters. Data were recorded in two 10-min intervals: one immediately before and one immediately after the intervention. Recordings were conducted with the participant seated, eyes open, and breathing spontaneously. The signal was visually inspected to ensure the absence of motion artifacts or arrhythmias. Only stable, artifact-free intervals were retained for analysis.

The HRV data were processed using Kubios HRV Premium software (v3.5, University of Eastern Finland, Kuopio, Finland), applying automatic artifact correction with a medium filter setting. Analysis was performed in both the time domain—reporting metrics such as the standard deviation of NN intervals (SDNN) and the root mean square of successive differences (RMSSD)—and the frequency domain, using Fast Fourier Transform (FFT) to extract power in low-frequency (LF: 0.04–0.15 Hz) and high-frequency (HF: 0.15–0.4 Hz) bands, expressed in both absolute (ms^2^) and normalized units (nu).

### 2.5 Intervention protocol

The intervention was administered by a licensed practictioner with advanced training and specialization in clinical functional neurology. The practitioner followed standardized protocols consistent with the ®NeuroReEvolution methodology and adapted them based on the patient’s individual neurological findings.

Functional neurology is a clinical approach that applies principles of neuroplasticity and sensorimotor integration to assess and modulate nervous system function through non-invasive stimuli (Escribano-Colmena et al., 2025). The intervention protocol was guided by the premise that specific sensory inputs—such as vestibular stimulation, oculomotor activation, and cervical mechanoreceptor engagement—can influence neural circuits involved in autonomic regulation (Escribano-Colmena et al., 2025a,b). This approach targets the central processing of afferent input with the goal of restoring functional balance between brainstem, cortical, and autonomic systems. In this case, the intervention was tailored based on the patient’s neurological findings and applied using brief, directed sensory-motor exercises aiming to stimulate adaptive brain responses (Rey-Mota et al., 2025a,b).

The intervention was tailored following a comprehensive clinical assessment of the participant’s neurological and psychological status, including verbal processing, visual tests for eye tracking and oculomotor reflexes, proprioceptive and motor coordination evaluations, and baseline HRV analysis to identify autonomic imbalances and guide intervention planning. This assessment revealed dysregulated autonomic function, reduced motor coordination, and psychological distress, informing the selection of neuromodulatory techniques (Rey-Mota et al., 2024a,b, 2025a,b).

The 45-min intervention followed a multimodal protocol grounded in functional neurology and based on ®NeuroReEvolution methodologies, designed to regulate autonomic function through sequential, individualized stimulation of sensory, visceral, and vestibular systems. The session began with a 15-min guided emotional recall and introspective exercise, during which the patient identified ten predominant dysfunctional emotions linked to previous traumatic experiences. This step was intended to activate the limbic system and facilitate top-down processing of emotional content.

The second phase focused on visuovestibular stimulation, targeting observed dysfunctions in saccadic control, convergence/divergence, and semicircular canal responsiveness. Controlled head movements and oculomotor exercises were used for approximately 8 min to recalibrate vestibular–brainstem circuits and improve visual–motor integration (Balaban and Yates, 2004; Shaffer and Ginsberg, 2017).

Subsequently, the patient’s previously identified lactose intolerance was addressed as a metabolic stressor potentially linked to emotional reactivity. This segment (5 min) involved specific visceral inputs to support regulatory responses in organs associated with emotional processing.

In the fourth phase, the practitioner applied manual stimulation to the visceral organs associated with the dysfunctional emotions previously identified. For approximately 10 min, neurovascular and neurovisceral reflex points were activated with the aim of modulating vagal tone and enhancing parasympathetic activity (Shaffer and Ginsberg, 2017).

Finally, the session concluded with blink reflex stimulation (2 min), targeting trigeminal–vagal pathways known to influence brainstem autonomic circuits, and short rest intervals were interspersed throughout the protocol to support neural assimilation and minimize sympathetic overload (Hammam and Macefield, 2017). Each component was carefully sequenced to maximize autonomic modulation and support a shift toward parasympathetic dominance (Table 1).

**Table 1:** Sequence and components of the functional neurology intervention.

Sequence order	Component	Approx. duration	Targeted system/objective
1	Guided identification of dysfunctional emotions (emotional recollection and prior trauma integration)	15 min	Emotional–limbic activation; preparatory regulation of cortical–visceral pathways
2	Visuovestibular stimulation (saccades, convergence/divergence, semicircular canals)	8 min	Brainstem and vestibular–autonomic integration; correction of oculomotor imbalance
3	Lactose intolerance activation and metabolic stress processing	5 min	Visceral–limbic axis; organ systems linked to emotional processing
4	Neurovisceral reflex stimulation of emotion-linked organs	10 min	Vagal tone modulation; neurovisceral reflex activation
5	Blink reflex activation	2 min	Trigeminal–vagal brainstem engagement; parasympathetic facilitation
—	Rest and assimilation periods between segments	5 min (interspersed)	Neural integration; autonomic rebalancing and fatigue mitigation

## 3 Results

Baseline psychological data, including measures of depression, anxiety, loneliness, psychological inflexibility, and perceived stress, were collected prior to the intervention to contextualize the patient’s emotional and autonomic state. These assessments were not repeated after the intervention, as the objective of the study was focused exclusively on physiological changes in HRV. Therefore, the psychological profile is presented as a descriptive reference to aid in interpreting the autonomic response, rather than as an outcome measure.

The participant sleeps 8 h per day, with her sleep quality over the past week rated at 4 out of 10. In terms of personality dimensions, she scores 4 in extraversion, 9 in agreeableness, 8 in conscientiousness, 5 in neuroticism, and 8 in openness to experience. Her psychological inflexibility, as measured by the AAQ II, is 14, while her loneliness score on the UCLA scale is 6. She also reports a moderate anxiety score of 16 on the STAI scale, a perceived stress score of 5 on the PSS-4, and a Zung Depression Scale score of 51, suggesting mild depression (Table 2).

**Table 2:** Results of psychological assessments of the participant.

Psychological test	Frequency (Items)	Score range	Participant score	Interpretation
Zung Depression Scale	20	20–80	51	Mild depression
Spielberger State–Trait Anxiety Inventory (STAI)	6	20–80	16	Moderate anxiety
UCLA Loneliness Scale (Short Version)	3	3–9	6	Moderate loneliness
Acceptance and Action Questionnaire-II (AAQ-II)	7	0–49	14	Moderate psychological inflexibility
Perceived Stress Scale (PSS-4)	4	0–16	5	Moderate perceived stress

Table 3 shows anincrease in heart rate (HR) by 10.1%, accompanied by a substantial rise in SD2 (230.5%) and HFnu (544.4%). In contrast, there were a decrease in SD1 (−52.4%), LF (−93.3%), and the LF/HF ratio (−93.0%).

**Table 3:** Modifications in heart rate variability data before and after the functional neurology intervention.

Variable	Pre	Post	% Change
HR (bpm)	69	76	10.1
SD1 (ms)	21	10	−52.4
SD2 (ms)	131	433	230.5
SD1/SD2	0.1603	0.0231	−85.6
LF (ms)	3,429	230	−93.3
HF (ms)	338	324	−4.1
LF/HF	10.14	0.71	−93.0
LF (nu)	91	42	−53.8
HF (nu)	9	58	544.4

HR, Heart Rate; SD1, Standard Deviation 1; SD2, Standard Deviation 2; SD1/SD2, Ratio between short-term and long-term heart rate variability; LF, Low Frequency; HF, High Frequency; LF/HF, Ratio of low to high frequency; LFnu, Normalized Low Frequency; HFnu, Normalized High Frequency.

## 4 Discussion

This case study investigated the immediate autonomic effects of a single-session, multimodal protocol derived from functional neurology principles. The intervention integrated several non-invasive techniques—including emotional recall, visceral stimulation, vestibular input, and oculomotor training—each targeting specific neural systems associated with autonomic regulation. Although the protocol was delivered in a single session, its layered structure and sequential combination of stimuli justified its classification as a multimodal intervention rather than a unitary treatment. This terminology more accurately reflects the complexity of the approach and supports the rationale for combining sensorimotor and neurovisceral techniques to acutely promote parasympathetic activation and reduce sympathetic dominance.

The participant presented with psychological symptoms—such as depression, anxiety, and emotional inflexibility—which have been associated in the literature with altered autonomic function. Rather than implying a unidirectional causal link, these factors were considered within a broader bidirectional framework, in which psychological distress and autonomic dysregulation may influence and reinforce each other through shared neurophysiological pathways.

The psychological tests in this case study highlight the participant’s emotional and mental health status. The Big Five assessment revealed high agreeableness (9/10) and openness to experience (8/10), traits linked to better emotional regulation and adaptability (Costa and McCrae, 1992), alongside moderate neuroticism (5/10), indicating vulnerability to emotional instability (Lahey, 2009). The Zung Depression Scale score of 51 suggests mild depression, consistent with findings of comorbid depressive symptoms in chronic pain patients (Bair et al., 2003). Moderate anxiety (STAI score of 16) aligns with studies linking chronic pain and anxiety (McWilliams et al., 2004). A perceived stress score of 5 (PSS-4) and UCLA Loneliness Scale score of 6 indicate moderate distress and loneliness, factors exacerbating autonomic dysregulation and mental health issues (Hawkley and Cacioppo, 2010). Psychological inflexibility (AAQ-II score of 14) reflects experiential avoidance, associated with greater distress and reduced well-being (Bond et al., 2011). These findings suggest moderate psychological distress contributing to autonomic dysregulation.

The techniques used in this intervention align with research on functional neurology and autonomic modulation, demonstrating potential to enhance parasympathetic activity and reduce sympathetic dominance (Shaffer and Ginsberg, 2017; Laborde et al., 2017). Vestibular and ocular reflex stimulation influence brainstem circuits, while proprioceptive and neurovisceral inputs support vagal tone and sensory-motor integration. Observed HRV changes in this study provide heuristic evidence for the efficacy of these non-invasive techniques, highlighting their relevance for autonomic regulation (Bustamante-Sánchez et al., 2020). However, the absence of post-intervention psychological assessments limits a full evaluation, emphasizing the need for further research with larger, controlled samples.

The participant’s heart rate increased a 10.1% increase post-intervention. While this might suggest an increase in sympathetic activity, heart rate alone is not a definitive marker of autonomic balance. Some studies indicate that interventions aimed at autonomic modulation can result in transient increases in heart rate, especially if parasympathetic function is being restored (Shaffer and Ginsberg, 2017). Thus, this rise in heart rate should be evaluated in conjunction with other markers such as SD1 and SD2, which reflect short- and long-term variability in heart rate. SD1, a measure of short-term HRV typically associated with parasympathetic activity, showed a sharp decrease of 52.4%. This reduction contrasts with the expected increase in parasympathetic tone following therapeutic interventions. However, SD2, which represents long-term HRV and is influenced by both sympathetic and parasympathetic activity, increased by 230.5%. This large increase in SD2 suggests an overall improvement in autonomic adaptability, even though short-term vagal tone (as indicated by SD1) was reduced. Such divergent trends between SD1 and SD2 have been observed in other studies where acute interventions lead to a temporary parasympathetic withdrawal, followed by a compensatory long-term autonomic stabilization (Task Force of the European Society of Cardiology, 1996; Bustamante-Sánchez et al., 2020).

The LF power, often linked to sympathetic activity, decreased by 93.3%, while the HF power, which is closely related to parasympathetic activity, showed only a slight reduction of 4.1%. This sharp decline in LF power and the relatively stable HF power suggest a significant reduction in sympathetic dominance, aligning with previous research showing that HRV improvements often correspond to reductions in LF power (Thayer et al., 2010). The LF/HF ratio, another critical indicator of the balance between sympathetic and parasympathetic activity, decreased by 93%, further emphasizing the shift towards parasympathetic predominance. Such a pronounced reduction in this ratio has been linked to improved emotional regulation and reduced stress levels (Kim et al., 2018). The normalized units of low frequency (Lfnu) and high frequency (Hfnu) also showed substantial changes. Lfnu decreased by 53.8%, while Hfnu increased by 544.4%. These changes reinforce the interpretation that the intervention significantly enhanced parasympathetic function. In similar studies, improvements in Hfnu have been associated with better autonomic resilience and a more balanced stress response (Laborde et al., 2017). The significant increase in Hfnu suggests that the functional neurology intervention led to a more favorable autonomic profile. While psychological assessments conducted pre-intervention suggest baseline distress, the lack of post-intervention psychological data prevents us from conclusively linking these changes to improved emotional regulation or psychological well-being.

In comparison to previous studies, the patterns observed in this case align with findings where therapeutic interventions aimed at modulating autonomic function led to increased parasympathetic activity and reduced sympathetic overdrive (Bellido-Esteban et al., 2022; Clemente-Suárez, 2020). The results, however, differ slightly in the magnitude of change in SD1, which may be attributed to individual variability or the specific methodology of the functional neurology intervention employed in this case. This suggests that while the overall trend of autonomic improvement is consistent with prior research, individual physiological responses can vary, and further studies are required to clarify these nuances.

The mechanisms through which functional neurology techniques may influence autonomic regulation are rooted in sensorimotor integration and neurovisceral connectivity. By delivering specific patterns of sensory input—such as vestibular, ocular, and visceral stimulation—these interventions are thought to activate afferent pathways that converge in the brainstem and project to autonomic control centers. In particular, stimulation of cervical mechanoreceptors, semicircular canals, and neurovisceral points may engage parasympathetic pathways via activation of the nucleus tractus solitarius and dorsal motor nucleus of the vagus. These circuits are associated with the modulation of dorsal vagal tone, which is linked to calm physiological states and improved autonomic flexibility. Furthermore, by reducing excessive sympathetic drive through reflex inhibition and sensory recalibration, the multimodal approach may contribute to an acute shift toward parasympathetic dominance, as reflected in the post-intervention HRV changes observed in this case (Escribano-Colmena et al., 2025a,b).

## 5 Limitations and future research

This study has several limitations. The lack of a placebo-controlled group makes it difficult to confirm that the HRV changes observed are solely due to the intervention. External factors such as diet, stress, and physical activity were not systematically controlled, potentially influencing the results. The study assessed only the immediate effects of a single session, limiting conclusions about the sustainability of HRV changes. The absence of post-intervention psychological assessments restricts understanding of the intervention’s emotional impact. Additionally, the single-case design limits generalizability. It is important to note that no psychological or symptom-based reassessments were conducted following the intervention. The study focused exclusively on physiological outcomes derived from HRV, and the absence of post-treatment psychological data limits conclusions regarding potential emotional or subjective improvements. Future research should include placebo-controlled designs, larger and diverse samples, and standardized protocols, alongside longitudinal studies to evaluate long-term effects.

## 6 Practical applications

The findings suggest that functional neurology interventions may be effective in improving autonomic regulation. However, further studies including pre- and post-intervention psychological assessments are necessary to determine their impact on emotional well-being. These techniques could be integrated into therapeutic protocols for conditions involving autonomic dysfunction and psychological distress, potentially offering a non-invasive alternative to conventional treatments.

## 7 Conclusion

This case study demonstrated that a single functional neurology intervention significantly improved autonomic modulation, evidenced by increased HFnu and decreased LF and LF/HF ratio, indicating a shift toward parasympathetic dominance. These findings align with prior research on non-invasive neuromodulation techniques and their potential to enhance autonomic balance. While limited by a single-case design, the study highlights HRV as a key outcome measure and supports functional neurology as a promising approach for managing autonomic dysfunction and psychological distress, warranting further investigation through larger, longitudinal studies.

## Data Availability

The datasets presented in this article are not readily available because of ethical and privacy restrictions. Requests to access the datasets should be directed to the corresponding author.
